# P300 Brain–Computer Interface-Based Drone Control in Virtual and Augmented Reality

**DOI:** 10.3390/s21175765

**Published:** 2021-08-27

**Authors:** Soram Kim, Seungyun Lee, Hyunsuk Kang, Sion Kim, Minkyu Ahn

**Affiliations:** School of Computer Science and Electrical Engineering, Handong Global University, Pohang 37554, Korea; soram523@gmail.com (S.K.); sokon459@gmail.com (S.L.); hsuk6032@gmail.com (H.K.); sionkim96@gmail.com (S.K.)

**Keywords:** brain–computer interface (BCI), virtual reality (VR), augmented reality (AR), P300, drone, BCI application, electroencephalogram (EEG)

## Abstract

Since the emergence of head-mounted displays (HMDs), researchers have attempted to introduce virtual and augmented reality (VR, AR) in brain–computer interface (BCI) studies. However, there is a lack of studies that incorporate both AR and VR to compare the performance in the two environments. Therefore, it is necessary to develop a BCI application that can be used in both VR and AR to allow BCI performance to be compared in the two environments. In this study, we developed an opensource-based drone control application using P300-based BCI, which can be used in both VR and AR. Twenty healthy subjects participated in the experiment with this application. They were asked to control the drone in two environments and filled out questionnaires before and after the experiment. We found no significant (*p* > 0.05) difference in online performance (classification accuracy and amplitude/latency of P300 component) and user experience (satisfaction about time length, program, environment, interest, difficulty, immersion, and feeling of self-control) between VR and AR. This indicates that the P300 BCI paradigm is relatively reliable and may work well in various situations.

## 1. Introduction

The brain–computer interface (BCI) is a technology that allows for direct communication between humans and a computer through the user’s brain activity and mental states [1]. A BCI can improve the quality of life by translating the brain activity into “mental commands” that can be exploited in a wide number of applications ranging from assisting people with disabilities or re-education therapies to entertainment and video games [2].

P300 BCI is one of the popular paradigms used in the BCI field. P300 is a positive event-related potential (ERP) that appears approximately 300 ms after the stimulus [3]. Farwell and Donchin introduced modern P300-based BCI in 1988 as a means of communication for patients suffering from the ‘locked-in’ syndrome [4]. The P300 Speller is a typewriter that uses the P300-based BCI paradigm. It consists of rows and columns with alphabetic and numeric characters; most are non-target and several are target stimuli. It detects the user’s intended character (target) based upon the P300 component elicited by flashing rows and columns [4]. The goal of the P300 is to analyze the ERPs in one or more repetitions to pinpoint which item has produced a P300, which is a positive ERP that appears approximately 300 ms after the item the user wants to select has flashed [5].

Many BCI studies in the past were based upon 2D visual stimulation but the recent development of head-mounted displays (HMDs) has paved the way to the commercialization of combined BCI+VR (virtual reality) or BCI+AR (augmented reality) [5]. Indeed, HMDs provide a build-in structure that can support the embedding of EEG (electroencephalography) electrodes, which are necessary for BCI [5].

With the emergence of HMDs, some BCI studies have been conducted that combine VR or AR using a HMD. Table 1 provides an overview of previous VR-BCI and AR-BCI studies. It summarizes the application contents, BCI paradigm, and display type. The AR systems were classified according to the type used: video-see through (VST) and optical see through (OST).

However, the development of BCI content in VR and AR environments has not been carried out actively because of the limited performance of BCI-based control, the two technologies’ inherent complexity, as well as the difficulty in designing effective UIs based upon BCI [2]. Furthermore, no BCI study has been conducted in both AR and VR to compare the performance in the two environments, although there are BCI studies that include either VR or AR. Therefore, it is necessary to develop a BCI application that can be used to compare the performance in both VR and AR and to test their feasibility in real-world environments.

To do so, we developed a BCI drone control application in this study that can be used in both VR and AR. In VR, a virtual drone was controlled with virtual maps, while in AR, a real drone was controlled and real-time video could be viewed with a drone camera. Using this application, we conducted an experiment with 20 subjects and compared the differences in the VR and AR environments.

In general, AR using head-mounted displays (HMD) includes optical see-through (OST) and video see-through (VST) [24]. The OST-HMD does not obstruct the subject’s front view and allows the user to observe the surrounding environment directly. The VST-HMD often captures environmental information through the camera so that the subjects can still obtain information about the surrounding environment. In this study, we use VR-assisted AR that floats virtual directional buttons above a real time video from a drone’s camera. Note that the AR we compare with VR in this study is video see-through (VST) AR.

To compare the performance in VR and AR effectively, we focused on one BCI paradigm (P300), two environments (VR and AR), and one task (mobile drone control).

We selected the P300 paradigm for our drone control system for the following reasons. First, drone control systems require rapid interaction between users and a drone, as the direction in which a user wants to go must be controlled with a rapid response with higher accuracy. Second, compared to the motor imagery BCI paradigm, P300-based BCIs require less training time and achieve a higher accuracy and information transfer rate (amount of information sent per unit of time). Third, P300 allows for a larger number of possible commands [18,25], which can be used as the directional buttons (forward, up, down, right, right turn, left, and left turn) in the drone control system. Finally, the P300 paradigm provides an intuitive user interface (UI); what a user sees is what should be recognized [26,27,28,29], which is essential to control the drone easily. In the SSVEP paradigm, the visual stimulus was flickered at different frequencies depending upon each target button, which can cause a different performance for each target button [30,31]. In addition, all target buttons in the P300 paradigm have the same ISI and ERPs corresponding to each target button and are preprocessed with the same procedure. Therefore, P300 was selected as the BCI paradigm for the drone control system to ensure that all directional buttons have a similar effect regardless of the order or frequency of the instructions because all instructions to control the drone were generated randomly in our experiment.

The first objective of this paper was to introduce an opensource-based drone control application using P300 BCI in the AR and VR environments. This application can allow people who are physically challenged to travel outside their normal environment and increase their autonomy by controlling a drone with their brainwaves alone. In addition, it also allows for more immersive experiences by combining BCI with VR and AR environments using a HMD.

The second objective of this paper was to study the feasibility of combining BCI and VR or AR technologies and compare the VR and AR environments. We evaluated the two environments from two perspectives: online performance and user experience. We compared the classification accuracy and analyzed the differences in event-related potential (ERP) signals in each environment. We also compared user satisfaction in the two environments and their effect according to preference.

## 2. Materials and Methods

### 2.1. Application Development

#### 2.1.1. Development Environment

In this project, we used OpenViBE, Python, Unity 3D, and Tello for Unity, which are competitive with respect to portability, scalability, and online performance.

OpenViBE for integration overall: This is an open-source software platform that is specialized in integrating various components of the brain–computer interface [32]. OpenViBE allows brain waves to be acquired, preprocessed, classified, and visualized in real-time and specializes in designing and testing brain–computer interfaces. In this project, we implemented the OpenViBE designer code for the Training Session and Online Session. These components collect brain signals from an EEG device (DSI-VR300, Wearable Sensing Inc., San Diego, CA, USA), the stimulation information from Unity, and save data in a file.

Python for signal processing: Signal processing algorithms were implemented in Python. Brainwaves were processed with Python to detect the P300 signal and the target button that a user saw was predicted by a pre-constructed classifier.

Unity 3D for VR and AR application: Unity 3D is a cross-platform game engine that can be used to create three-dimensional, VR, and AR games, as well as simulations and other experiences [33]. In this project, it was used to display a visual panel that blinks randomly, virtual maps for drone movement, and video from a drone on the screen.

Tello for Unity to control the drone: Unity API was used to control a DJI Tello drone in the AR mode [34]. This API provides a function to move the drone in several directions, automatic take-off and landing functions, as well as hijack functions to control the drone with keyboards in case of emergency [34]. It also allows users to view real-time video from the drone on screens in the HMD [34]. However, Tello for Unity uses a keyboard to control drones by giving them constant commands. To address this, we made it possible for the drones to travel a certain distance with a single command by relaying commands continuously over a period of time.

All the source codes for the drone control system are available through the Github repository [35].

#### 2.1.2. System of Drone Control Application

**Figure 1:**
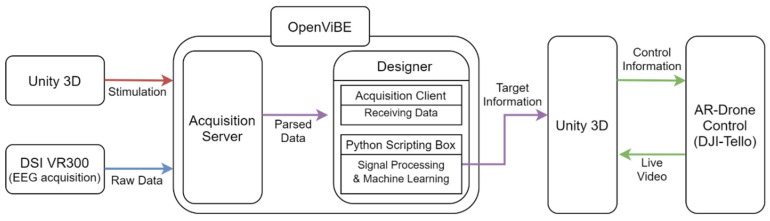
The structure of the drone control system overall.

The drone control system’s environment and composition are shown in Figure 1. We implemented an application to control the drone in the AR and VR environments using Unity. In Unity, an interface for blinking buttons was implemented to provide visual stimulation. Unity relays the timing information of a flickering button to OpenViBE through the TCP/IP protocol. OpenViBE parses the timing information from Unity and the data from DSI-VR300 into a single file. The timing information and EEG signal are processed in Python to predict the user’s direction to control the drone. The command predicted is transferred to either a virtual or real drone. A real drone transfers the real-time video from the drone camera back to Unity through Wi-Fi.

#### 2.1.3. Signal Processing and Classification

**Figure 2:**
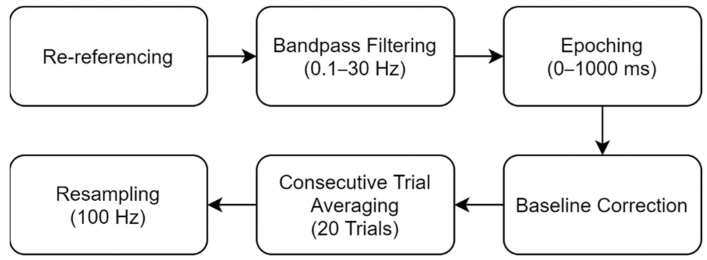
Signal processing procedure.

Basically, the signal acquired was processed through a pre-defined pipeline, as shown in Figure 2. Each step is described as follows.

- Re-referencing: seven channels (Fz, Pz, Oz, P3, P4, PO7, and PO8) were re-referenced using a channel on the left ear.
- Bandpass filtering: data were filtered to the frequency band of 0.1–30 Hz using the SciPy package with the 5th order Butterworth filter [36].
- Epoching: data were segmented to 0–1000 ms epochs from each stimulus onset.
- Baseline Correction: Each epoch’s mean value was subtracted from the epoch. EEG signals are prone to amplitude shifts attributable to such factors including changes in impedance or noise, which can be fatal in the data analysis. Baseline correction compensates for this random amplitude shift.
- Consecutive Trial Averaging: to improve the signal-to-noise ratio (SNR), segmented epochs were averaged continuously by 20 epochs.
- Resampling: Brain signals were digitized originally at a sampling rate of 300 Hz. To reduce the data size, signals were down-sampled to 100 Hz.

The EEG data acquired during the training session were processed through the steps in Figure 2 and a classifier was constructed using these data. Then, this classifier was used in the following two online sessions. Linear discriminant analysis (LDA) was used as our classifier. LDA uses a linear hyperplane to separate data from two classes, assuming that data are distributed normally [37]. As its classification is highly efficient despite its simplicity, this classifier is used widely for BCI designs, particularly for P300-based BCI [38]. The EEG data from the online session were processed through the same steps in Figure 2. All outputs from the classifier across blinks were summed and the selection (direction button) with the highest value was chosen as the target direction.

#### 2.1.4. Game Scenario and Contents

**Figure 3:**
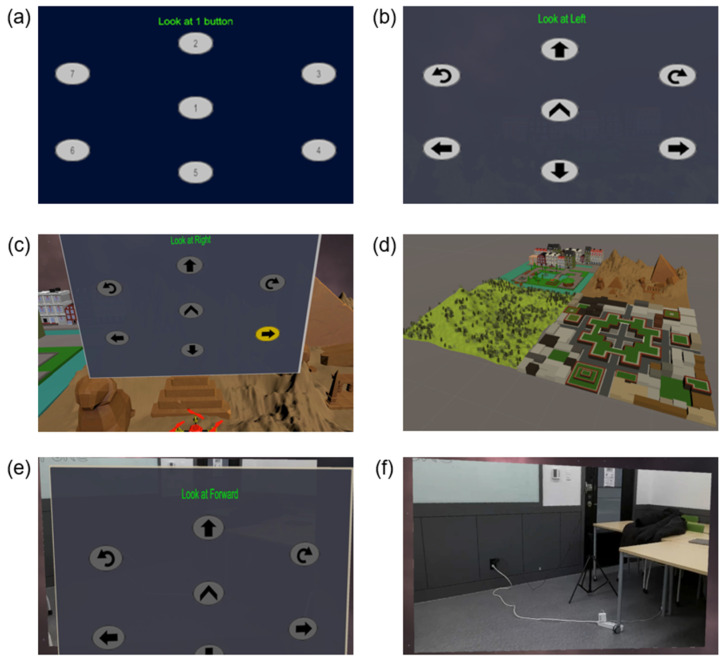
UI of the system developed. (**a**) Button interface for the training session. (**b**) Button interface for the online session. (**c**) VR content to control a virtual drone. Desert map is shown. (**d**) All four maps (park, dessert, maze, and forest). (**e**) AR contents to control a real drone. Button interface is shown. (**f**) A real video in HMD streamed from the drone.

The characteristics of the P300 component in an ERP may differ in appearance, latency, and amplitude depending upon users’ individual differences, age effects, and ultradian rhythms [39]. Thus, it is reasonable to collect data during a training session before running a real-time BCI application (e.g., testing, or online session). As most BCI systems, the application developed consists of two phases: the training session and online session. Thus, we designed a training scenario as shown in Figure 3a and then the classifier was used to detect the target button from Figure 3b, which is the UI during the testing session.

In the training session, the user looks at the target button according to the instructions on the top. In one selection of a training session, seven buttons blink 30 times with a fixed ISI in random sequence. “Blink” in this system refers to visual stimulation using a color that toggles between gray and yellow. A button toggles to yellow for 100 ms and re-toggles to gray for 100 ms to give one visual stimulation.

After the training session, online sessions are run to control the drone. The button interface for online sessions is shown in Figure 3b. There are seven directional buttons (forward, up, down, right, right turn, left, and left turn). In one selection of the online session, seven buttons blink 20 times with fixed ISI in random sequence. The user looks at the button in the direction she/he wants to move the drone and this application predicts the direction the user is looking at and controls the drone.

**Figure 4:**
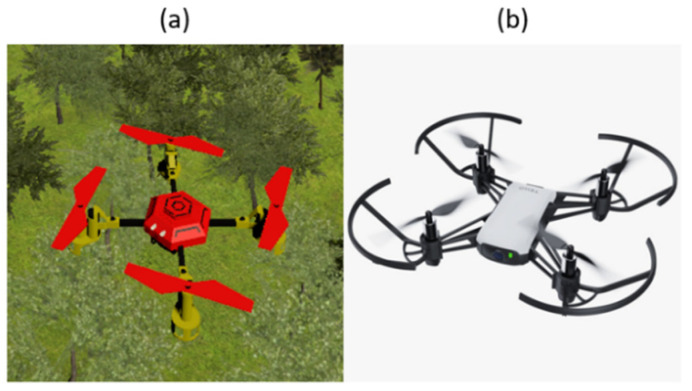
Images of a drone used in this system: (**a**) a drone used in the virtual environment and (**b**) a drone used (DJI-Tello) in the real environment.

**Figure 5:**
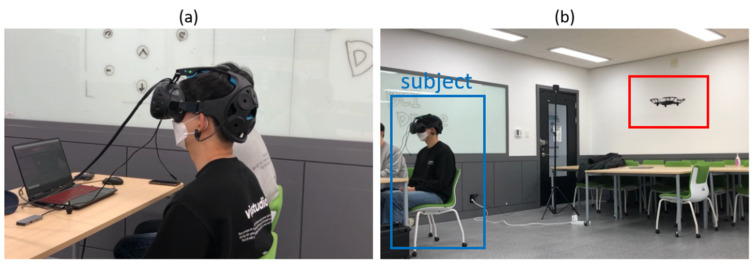
Experimental view. (**a**) A subject wearing HMD is playing the drone control system. (**b**) A real drone (red box) is being controlled by a subject (blue box) through the AR-drone control system.

The drone control application is divided into VR and AR contents depending upon the environment. Figure 3c,d are parts of the VR application with which a user controls a drone in the VR environment. In the VR environment, the virtual drone (Figure 4a) allows the user to navigate a virtual map of his/her choice (park, desert, maze, and forest). Figure 3e,f are images of controlling a drone in the AR environment. Furthermore, in the AR environment, a drone (Figure 4b) can be controlled in real environments through a real-world drone (DJI-Tello) (Figure 5b) and users can watch real-time images from the drone’s camera.

### 2.2. Experiment

#### 2.2.1. Subjects

To compare the performance in VR and AR, 20 subjects (age: 20 to 27, ten males and ten females) participated in this experiment with the same equipment in the same place and performed one task (mobile drone control) in VR and AR sequentially. The Public Institutional Bioethics Committee designated by the Ministry of Health and Welfare approved the study (P01-201812-11-004) and all subjects were informed about the experiment and their right to stop the experiment if/when they felt uncomfortable or experienced 3D motion sickness before the experiment. All subjects signed a written consent form prior to this experiment and were paid for their participation. None of them had participated in a BCI experiment before this study.

**Figure 6:**
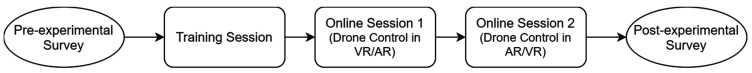
Experimental procedure. Half of the participants engaged in the online sessions in the order of AR followed by VR and the other half engaged in the order of VR followed by AR.

Each experiment took a total of 50 min. The experimental procedure is shown in Figure 6. Before and after the experiment, the experimental survey was conducted. Training sessions were conducted to generate a classifier, followed by online sessions to control the drone. Half of the participants engaged in the AR online session after the VR online session and the other half engaged in the VR online session after the AR online session. To test their accuracy in the online session, the subjects were instructed to move the drone according to the instructions given at the top in the display. Each subject played 15 selections (15 in VR and 15 in AR) in two online sessions.

The EEG data were recorded with DSI-VR300, which has a HMD (HTC-Vive) and a headset to measure the EEG (Figure 5a) [40]. The device has seven channels of dry electrodes (Fz, Pz, Oz, P3, P4, PO7, and PO8) and a 300 Hz sampling frequency [40]. A DJI-Tello drone was used in the AR applications (Figure 4b and Figure 5b) [41].

#### 2.2.2. Questionnaire

Two questionnaires were generated pre and post-task (Table 2). Before the experiment, the subjects filled out a pre-task questionnaire that includes questions about mental disease, experience with BCI content or AR and VR contents, and 3D sickness. They were also asked of their condition, such as the number of hours they slept and hours elapsed since they had ingested alcohol, coffee, or cigarettes. After the experiment, the subjects filled out a post-task questionnaire about their satisfaction with VR and AR to compare the two environments. They evaluated their satisfaction with the time, program, environment, interest, difficulty, immersion, feeling of self-control of the drone, and 3D sickness. They also indicated their predicted accuracy from 1 to 10 as well as their preferred environment (VR or AR) and reason for preference.

#### 2.2.3. Analysis and Statistical Tests

Primarily, we investigated the online performance, peak latency, and amplitude of the ERP component to compare the VR and AR environments. The scores and answers to the questionnaires were also examined to assess any differences between the two. We used the Wilcoxon signed-rank tests to analyze the data, as the Kolmogorov–Smirnov test indicated that most of the data did not follow a normal distribution. We set the significance level to 0.05.

## 3. Results

Before the analysis of the experimental results, we looked at all subjects’ full P300 waves in 30 selections (15 each in VR and AR) one by one. In one selection, there was 20 epochs (or blinks) per button. In total, 140 epochs (20 epochs × 7 buttons) were required to predict one directional button. If the maximum amplitude of the ERP in each epoch exceeded 80 μV, which is over the conventional range of P300 amplitude [42,43], the epoch was considered poorly measured, and if these exceeded 50% of the total epochs, the selection was considered to be noise-contaminated. If at least 10 of the 15 selections (66%), either in AR or VR, were considered noise-contaminated, the subject was excluded from the analysis. As a result, three subjects (S07, S12, and S13) were excluded from the analysis because of heavy noise in their EEG signals.

Performance in each environment, 3D motion sickness, and environment preference are shown in Table 3. We compared the VR and AR environments from two perspectives: online performance (classification accuracy and ERP signal) and user experience (satisfaction and preference) in Section 3.1 and Section 3.2.

### 3.1. Online Performance in AR and VR

#### 3.1.1. Accuracy in VR and AR

**Figure 7:**
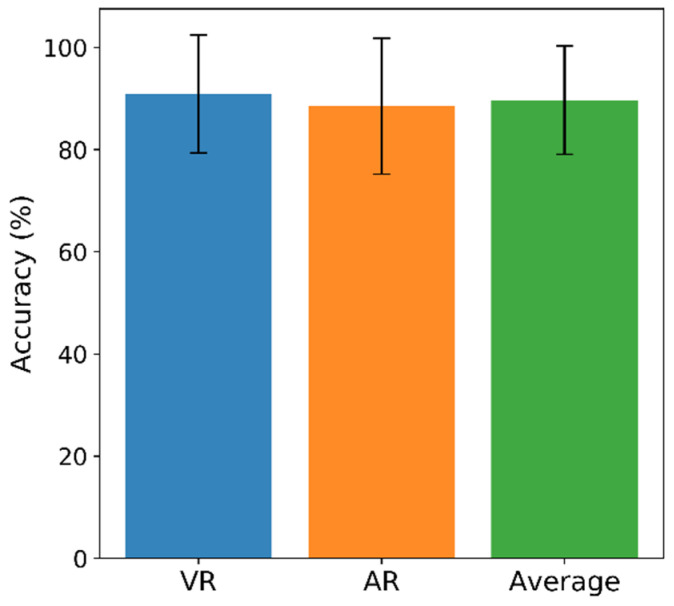
Mean accuracy and standard deviation are presented for VR (blue), AR (orange), and the mean of VR and AR (green). No significant difference was observed (*p* > 0.05).

The classification accuracy was calculated to evaluate the two-drone control system. Figure 7 shows the subjects’ accuracy in VR and AR and the mean. On average, the subjects’ performance was 90.88% (VR) and 88.53% (AR), with a mean of 89.71% overall. The Wilcoxon signed-rank tests were performed to test the statistical significance and showed that the classification accuracy did not differ significantly in the two environments (*p* > 0.05).

#### 3.1.2. ERP in AR and VR

To compare the difference in ERP signals in the two environments, we analyzed the latency and amplitude of the peak in the P300. In this research study, AR for comparison with VR is VR-assisted and video see through-based. Three-hundred epochs (15 selection × 20 blinks) in one environment were averaged per subject; we identified the peak of the P300 component in the averaged ERP manually and calculated the peak value’s amplitude and latency.

As shown in Table 4, the mean latency in VR and AR were 415.88 ± 22.77 ms and 411.76 ± 23.82 ms, respectively, and the mean amplitude in the two were 5.09 ± 4.79 µV and 6.22 ± 7.94 µV, respectively.

The Wilcoxon signed-rank tests were used to test the statistical significance of the ERP because the Kolmogorov–Smirnov test showed that the latency and amplitude dataset also did not follow a normal distribution. The Wilcoxon signed-rank test showed that the latency and amplitude of the P300 peak point did not differ significantly in the VR and AR environments (*p* > 0.05).

In the analysis above, each subject’s mean target ERP was compared in VR and AR. This time, we divided the targets into seven directions (forward, up, down, right, right turn, left, and left turn) and analyzed the ERP signal depending upon the directions.

**Figure 8:**
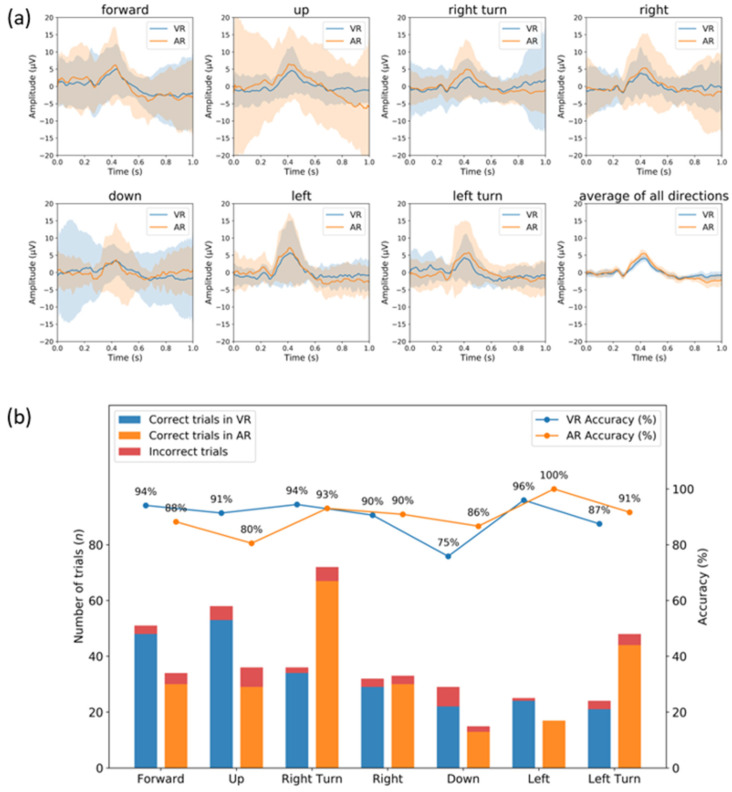
(**a**) ERP signal (*n* = 17) averaged over seven directions (forward, up, down, right, right turn, left, and left turn) in VR (blue line) and AR (orange line) environments. Standard deviations are presented with areas. (**b**) All subjects’ direction-based accuracy.

Figure 8a shows the averaged ERP of the seven directions between 0 and 1000 ms after the stimulus. The ERP signal of 17 experiments in VR and AR were averaged in each direction.

As observed, prominent peaks presented at approximately 0.4 s for both VR and AR. To examine the difference in peak amplitude between the two environments, we collected the mean amplitude from 0.3 to 0.5 s in each direction and conducted a statistical test (Wilcoxon rank-sum test). However, we found no statistically significant difference between the AR and VR peak amplitudes in all directions (*p* > 0.05, FDR corrected [44,45]).

Furthermore, we collected all trials from all subjects and attempted to calculate all subjects’ direction-based accuracy. Figure 8b presents the results. Overall, direction-based accuracies were 75% (minimum) to 95% (maximum) for VR and 80% to 100% for AR. However, no statistically significant difference was found between the two environments (*p* > 0.05).

### 3.2. User Experience in AR and VR

We also evaluated the users’ experience using the answers from the two questionnaires in Table 2, alongside any difference associated with VR or AR preference and 3D sickness.

#### 3.2.1. Satisfaction

**Figure 9:**
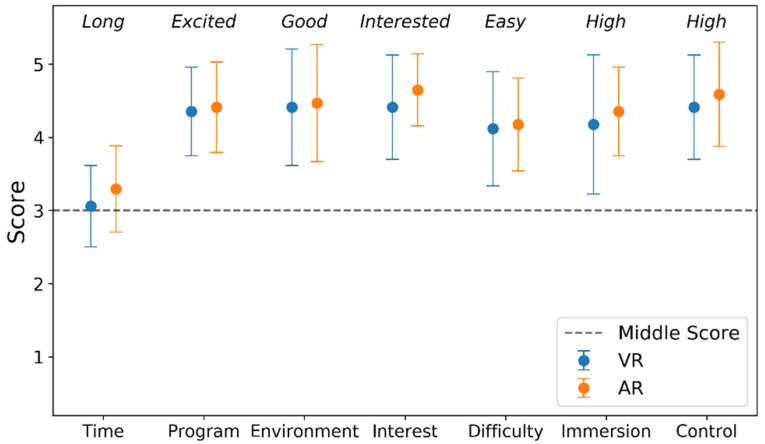
Users’ satisfaction with VR and AR.

To compare the users’ satisfaction in VR and AR, seven items (time length, program, environment, interest, difficulty, immersion, and feeling of self-control) were evaluated. Each item was scored from one to five points on VR and AR and three points indicates moderate or neutral. The mean score and standard deviation for the seven items evaluated are shown in Figure 9. The Wilcoxon signed-rank test showed that the users’ experience of the seven items did not differ significantly in the VR and AR environments (*p* > 0.05).

#### 3.2.2. Accuracy According to Preference

**Figure 10:**
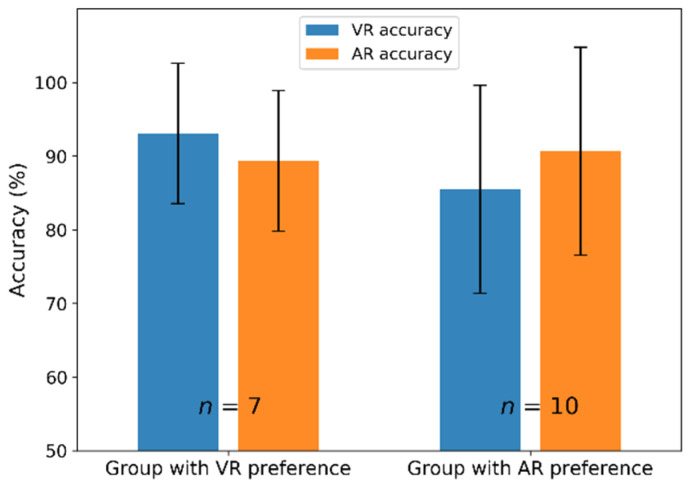
Accuracy according to the environment preferred.

The mean accuracy between VR and AR did not differ significantly in Section 3.1.1. However, the accuracy differed slightly depending upon the subjects’ preferred environment. Figure 10 shows the group’s mean accuracy according to preference. The mean accuracy in the group that preferred VR (*n* = 7) was 93.1% in VR and 89.33% in AR, which indicates that VR accuracy was slightly higher than that in AR. In addition, the group that preferred AR (*n* = 10) showed greater accuracy in AR (90.67%) than VR (85.48%). However, the statistical test showed no significant difference between the two (VR preferred: *p* = 0.23336, AR preferred: *p* = 0.58091).

#### 3.2.3. User’s Self-Predicted Performance

**Figure 11:**
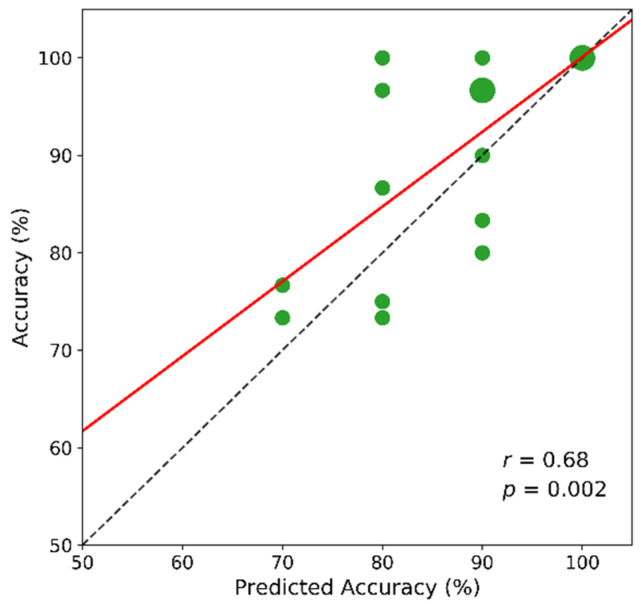
Actual accuracy and users’ self-predicted accuracy. The circle size represents the number of subjects in that point. The dashed line is the *y = x* line and the solid red is the regression line on the given points.

BCI users’ self-prediction of their accuracy is useful information for understanding BCI performance variation [46,47,48,49] and there is typically a high positive correlation between self-predicted BCI performance and actual classification accuracy [47]. Similarly, in this drone control experiment in VR and AR, the subjects were asked to predict their accuracy overall after the experiment. Figure 11 presents the predicted and actual accuracies averaged over the two environments. Interestingly, we also found that their self-predicted accuracy was correlated highly positively with their actual classification accuracy (*r* = 0.68, *p* < 0.05).

## 4. Discussion

In this study, we demonstrated a P300 BCI drone control application for VR and AR environments and showed that the two environments did not differ significantly in performance (classification accuracy and ERP patterns) and user experience (time length, program, environment, interest, difficulty, immersion, and feeling of self-control).

We extended the conventional 2D-based BCI experiment to a 3D-based BCI experiment in both virtual reality (VR) and augmented reality (AR) using a HMD. A wearable HMD-based BCI is promising as it provides a more user-friendly BCI system for hands-free interaction with real and virtual objects in AR and VR environments [50]. These 3D environments can be highly interactive and can provide a suitable way to alleviate the limitations of BCI control, such as slow and error-prone interactions and limited degrees of freedom [51]. Beyond the typical BCI paradigm, we developed the drone control application, which can be used in a real situation. This application can improve the level of freedom and offer the possibility for an immersive scenario through induced illusions of an artificially perceived reality that can be used not only in basic BCI studies but also in many other fields of application [52]. We also used dry rather than wet EEG electrodes because of their easy use in real situations for a user-friendly system.

In BCI studies, the user’s experience is one of the most important aspects of the system’s feasibility. We compared the two environments using not only performance but also users’ experience in a real-world environment and all participants showed a positive response in both VR and AR. This confirms the feasibility of applications available in both VR and AR with respect to users’ satisfaction as well as performance.

This work encourages more exciting and promising new research topics to further develop the association between BCI and mixed reality, which uses both VR and AR. In addition to the P300 paradigm, other paradigms, such as motor imagery and SSVEP, can also be tested in a mobile drone control system in VR and AR for more practical applications.

Both VR and AR offer immersive and interactive effects but even when the same HMD is used, some elements such as UI and contents can lead to different user satisfactions. Thus, we investigated the participants’ preferred environment as well as their satisfaction.

Although the subjects expressed the same positive opinion about the two environments, they had their own preferred environment and corresponding reasons. The reasons why some preferred VR were “interest in a virtual environment” and “feeling of playing the game”, while the reasons why others preferred AR were “strong immersion due to the AR”, “controlling of real drone”, and “liveliness of real time video”.

Section 3.2.2 showed that classification accuracy was slightly higher in the subjects’ preferred environment. However, there was no statistically significant difference between VR and AR accuracy in either the group that preferred VR (*n* = 7) or AR (*n* = 10). In general, a small sample size can influence statistical tests’ significance [53] and a larger sample increases reliability and minimizes the effect of measurement error [54]. Thus, we expect that more data would help us draw a solid conclusion about the relation between environment preference and BCI performance. Furthermore, there is another possible scenario. Subjects may prefer an environment in which they believe their accuracy is higher, i.e., perceived accuracy may affect the environment. However, in this study, we surveyed the subjects’ environment preference after an experiment. One possible approach to check whether there is bias attributable to the experience (regarding accuracy) would be to provide the VR and AR experience without classification feedback and conduct a post-experimental survey. In the future, we will consider this when designing an experiment.

Now we discuss the potential limitations and issues with respect to user-friendly BCI applications. As in traditional ERP-based systems, this drone control application requires a training session before use. During the session, a classifier for the online session is constructed by applying a supervised algorithm to adjust the user’s ERP signal [55]. However, throughout this process, the subject is instructed to focus on a specific direction button without any meaningful interaction. This calibration process is one of the major factors that limits the current BCI systems’ progress [56]. Therefore, to achieve a practical BCI system, we need to develop a BCI application that requires no calibration.

Various studies have been conducted to minimize or reduce the calibration process. Lotte et al. proposed regularized canonical correlation analysis combined with a regularized linear discriminant analysis in 2009 [57], and in 2011, Rivet et al. proposed an adaptive training session to reduce the calibration time [58]. Recently, the convolutional neural network (CNN) has been proven to be useful in optimizing EEG classification [59,60] because of the model’s ability to capture local features, which should be sufficiently robust to data shifts [61]. An ideal calibration-free algorithm should follow one of two paths: (1) determine a set of generalized features that do not differ among subjects or (2) store various forms of P300 signals for comparison [56]. In the future, we will investigate various models (such as the CNN, random forest, and ensemble classifier) that fit our system and collect a large sample of ERP data in VR and AR. Ultimately, we will develop a user-friendly BCI drone control application that requires no training session.

Another aspect to improve in our experiment is to include an evaluation of the drone’s free flight through user’s intention. In this experiment, the subjects had to move the drone according to designated instructions to test their accuracy. If the result of a user’s P300 classification matched the specified instruction, the drone moved in that direction; otherwise, it did not. In fact, controlling the drone in the direction that the user wants it to go is one of the important points in this system. Therefore, in the future, we need to test the drone’s free flight performance through users’ intention.

The last issues are related to the basic concept of this study. Many P300 BCI applications have been implemented in a desktop environment (2D monitor screen). Hence, a desktop-based BCI application may provide a different user experience. In this study, we only compared VR with AR. Thus, investigating any differences in user feedback in the three environments would provide insightful information that may be helpful in designing a new BCI application. Furthermore, different control paradigms (e.g., SSVEP or motor imagery) may provide different experiences as well. Considering the methods used to command vary somewhat across control paradigms, VR and AR should be tested in different BCI types as well. Finally, we recruited 20 subjects and used data from only 17. Although this is not a small sample, more data will help strengthen the results, particularly for group analysis (VR/AR preferred or 3D sickness, etc.). In the future, we will consider these issues in further studies.

## 5. Conclusions

We developed a P300 BCI drone control application that worked with VR and AR. To check the difference between and influence of the different environments, an online experiment was conducted with 20 subjects and their opinions were collected using a questionnaire. We found that all subjects controlled a drone in the VR and AR environments well and expressed the same positive opinions about both environments. These results demonstrate that VR and AR do not differ in performance (including ERP patterns) and user experience. Thus, we conclude that the P300 BCI paradigm is relatively stable and works well in various situations.

## Figures and Tables

**Table 1:** Overview of previous VR-BCI and AR-BCI studies. Motor imagery (MI) and steady-state visual-evoked potential (SSVEP).

Environment	Application Contents	Study	BCI-Paradigm	Display Type/ (AR Type)
VR	Post-stroke rehabilitation	Aamer et al. [6]	MI	HMD
	Attention training	Mercado et al. [7]	Neurofeedback	HMD
	BCI system	McMahon et al. [8]	MI	HMD
	Attention training	Rohani et al. [9]	P300	HMD
	Attention training	Ali et al. [10]	SSVEP	HMD
AR	Real-time monitoring applications	Arpaia et al. [11]	SSVEP	HMD/OST
	Robot-based rehabilitation	Arpaia et al. [12]	SSVEP	HMD/OST
	Robot control	Si-Mohammed et al. [2]	SSVEP	HMD/OST
	Home appliance control	Park et al. [13]	SSVEP	HMD/OST
	Quadcopter control	Wang et al. [14]	SSVEP	HMD/VST
	Communication	Kerous et al. [15]	P300	HMD/VST
	Robotic arm control	Zeng et al. [16]	MI	CS/VST
	Robot control	Tidoni et al. [17]	P300	HMD/VST
	Wheelchair control	Borges et al. [18]	SSVEP	HMD/VST
	Feasibility study	Bi et al. [19]	P300	head-up display
	Robot control	Martens et al. [20]	P300, SSVEP	HMD/VST
	Light and TV control	Takano et al. [21]	P300	HMD/OST
	Robot control	Faller et al. [22]	SSVEP	HMD/VST
	Robotic arm control	Lenhardt et al. [23]	P300	HMD/VST

**Table 2:** Pre- and post-questionnaire items.

Questionnaires	Question	Answer Format
Pre	Do you have mental disease?	Yes or No
	Have you ever participated in a BCI experiment?	Yes or No
	Have you ever experienced AR or VR contents?	Yes or No
	Have you ever experienced 3D motion sickness?	Yes or No
	Did you sleep well for more than 6 h?	Yes or No
	Did you drink coffee within 24 h?	Yes or No
	Did you drink within 24 h?	Yes or No
	Did you smoke within 24 h?	Yes or No
	Evaluate your physical condition.	1 to 5 (good)
	Evaluate your mental condition.	1 to 5 (good)
Post (VR and AR)	Evaluate the playing time (time).	1 to 5 (long)
	Evaluate how you feel about this application (program).	1 to 5 (excited)
	Evaluate the comfort of surroundings (environment).	1 to 5 (good)
	Were you interested in the application (interest)?	1 to 5 (interested)
	Was the application difficult (difficulty)?	1 to 5 (easy)
	Evaluate the immersiveness of the application (immersion).	1 to 5 (high)
	Evaluate the ability to control the drone (control).	1 to 5 (high)
	Did you feel 3D motion sickness?	Yes or No
	Please predict your performance.	1 to 10 (high)
	What do you prefer, VR or AR?	VR or AR

**Table 3:** Results of the experiment.

Subject No.	Accuracy (%)			3D Sickness		Preference
	VR	AR	Mean	VR	AR	
S1	90.00	60.00	75.00	N	N	VR
S2	66.67	80.00	73.33	Y	N	AR
S3	95.00	85.00	90.00	N	N	VR
S4	80.00	73.33	76.67	N	N	AR
S5	80.00	66.67	73.33	N	Y	AR
S6	100.00	100.00	100.00	N	N	AR
S8	100.00	100.00	100.00	N	N	AR
S9	73.33	86.67	80.00	N	N	AR
S10	73.33	93.33	83.33	N	N	VR
S11	93.33	100.00	96.67	N	N	VR
S14	100.00	73.33	86.67	N	N	VR
S15	100.00	100.00	100.00	N	N	AR
S16	100.00	93.33	96.67	Y	Y	VR
S17	100.00	100.00	100.00	N	N	AR
S18	100.00	93.33	96.67	N	Y	VR
S19	93.33	100.00	96.67	N	N	AR
S20	100.00	100.00	100.00	N	N	AR

**Table 4:** Comparison of the ERP in VR and AR. Mean and standard deviation (SD) are presented at the bottom.

Subject No.	Latency (ms)		Amplitude (μV)	
	VR	AR	VR	AR
S1	410	420	5.65	6.05
S2	390	410	3.7	2.16
S3	390	380	2.72	4.97
S4	420	410	2.49	2.54
S5	440	430	9.05	5.28
S6	460	440	4.35	7.72
S8	420	410	3.8	4.74
S9	420	420	7.19	5.62
S10	400	400	2.25	2.94
S11	430	430	2.94	3.09
S14	370	340	4.48	9.01
S15	400	400	3.17	3.1
S16	420	430	1.47	2.24
S17	410	400	3.89	2.76
S18	450	430	3.59	2.86
S19	400	410	22.85	37.05
S20	440	440	2.88	3.53
Mean	415.88	411.76	5.09	6.22
SD	22.77	23.82	4.79	7.94

## Data Availability

The data presented in this study are available on request from the corresponding author.
